# Older Adults’ Response to Color Visibility in Indoor Residential Environment Using Eye-Tracking Technology

**DOI:** 10.3390/s22228766

**Published:** 2022-11-13

**Authors:** Ze-Yu Wang, Ji Young Cho

**Affiliations:** Department of Housing & Interior Design (AgeTech-Service Convergence Major), Kyung Hee University, Seoul 02447, Republic of Korea

**Keywords:** color visibility, eye-tracking, indoor residential environment, older adults

## Abstract

Older adults spend relatively more time in indoor residential environments than young people. As their visual and cognitive abilities decline, they experience a high risk of accidents in indoor environments; thus, understanding their gaze behavior while considering cognitive capacity is essential in preventing potential accidents and planning for aging-friendly environments. The purpose of this study was to examine whether the use of color in living environments affects the visual attention of older adults. The following two experiments were conducted using eye-tracking technology: Experiment 1: Older adults’ gaze behaviors were measured while viewing four images consisting of nine sets of door–door frames with different color combinations of safety colors, black and white; and Experiment 2: Based on results from Experiment 1, images of simulated living environments were created, and older adults’ gaze behaviors were measured while viewing eight images in which two door colors were applied to verify spatial color visibility. Their cognitive state represented by the Mini-Mental State Examination (MMSE) was then compared with their gaze behavior. The results show that: (a) participants paid more attention to doors with color combinations including red (red–black and white–red) and remained longer on the red–black combination; (b) older adults looked at red–black doors faster than white doors in the same position; (c) the dwell time on a red–black door was longer than on that of the corresponding position of a white door; and (d) based on participants’ MMSE values, the gaze behaviors of the group with low cognition were less focused and observable than those of the group with normal cognition. The results of this study are significant in that they reveal that indoor color can improve the visibility of elements that can impact safety in the living environments of older adults and enhance their quality of life.

## 1. Introduction

### 1.1. Research Background

With a low fertility rate and high life expectancy, the world’s population is aging. Globally, the population aged 65 years and over is growing faster than all other age groups. According to data from the World Population Prospects in 2019, one in six people in the world will be over the age of 65 by 2050, up from one in eleven in 2019. The number of persons aged 80 years or over is projected to triple, increasing from 143 million in 2019 to 426 million in 2050 [1]. As people grow older, they tend to spend more time in indoor living environments than young people, especially after retirement; thus, paying more attention to the appropriate design of indoor environments suitable for the aging population is essential [2].

Aging results in physiological changes to various sensory organs. The eye is the first organ that encounters the outside environment and, when affected by aging, problems such as poor vision, vitreous opacities, maculopathy, and narrowing of vision occur. The color sense of older adults changes and the lens ages. In addition, cognitive activity in the brain decreases to some extent with age [3,4]. Visual aging and cognitive decline adversely affect a person’s judgment of objects in residential spaces, especially in those who show symptoms of cognitive impairment. They can experience inconvenience due to problems with color discernment and narrow vision, and the risk of accidents tends to increase along with cognitive decline [5].

Eye-tracking technology can capture gaze behavior comfortably while the participant remains unaware of it, so little to no psychological or physiological discomfort occurs for older adults. The aim of the current study was to conduct experiments to understand older adults’ gaze behavior toward color visibility in a residential environment using eye-tracking technology.

We assumed that use of high color visibility in environments will help older adults understand the environment and reduce information processing time, especially for those who experience the decline of visual and brain function, improve the safety of their living environment and improve their quality of life, ultimately reducing the burden on their families brought about by their decreasing capacities to care for themselves.

### 1.2. Purpose and Significance

The purpose of this study was to investigate the effect of color visibility in indoor environments on older adults using eye-tracking technology and to provide guidelines for interior design for older adults in the future.

In order to understand the global research trend on this topic, we searched the Web of Science with eye-tracking-relevant keywords, older adult-relevant and environment-relevant keywords for research papers on older adults and spatial color using eye-tracking and obtained six results. Among them, only one study (“Effect of environmental factors on how older pedestrians detect an upcoming step” by Cheng et al., 2018) [6] fit the three conditions of eye-tracking, older adults and the environment, and included a focus on the relationship between safe mobility and environmental characteristics for older adults. Cheng et al. (2018) reported that shorter detection distance, a greater number of fixations, and longer fixation duration were found among older participants compared to young participants. The results suggested that the processing efficiency for visual information on an upcoming step was slower among older adults than young people [6].

When searching for eye-tracking, older adult-relevant keywords with the addition of color-related keywords, we obtained 44 results. Only four papers seemed relevant, including those on path map (“User typology based on eye-movement paths“ by Popelka et al., 2013) [7], contrast of obstacles in spaces (“Enhanced obstacle contrast to promote visual scanning in fallers with Parkinson’s Disease: role of executive function“ by Alcock et al., 2020) [8], difference in the effect of stair decoration on the vision of the older and young adults (“Influence of step-surface visual properties on confidence, anxiety, dynamic stability, and gaze behavior in young and older adults“ by Thomas et al., 2021) [9], and wayfinding in senior homes (“Wayfinding in aging and Alzheimer’s disease within a virtual senior residence: Study protocol“ by Davis et al., 2016) [10]. No eye-tracking studies have been conducted on the color of residential environments for older adults.

Therefore, given the lack of prior studies, this study has significance in exploring whether the change in color in residential spaces affects the visual attention of older adults. The significance of this study is that it can provide foundational knowledge of spatial color visibility for age-friendly environments by revealing older adults’ gaze behavior. Visual attributes according to the color change of the door and the door frame and surrounding environment in the indoor residential environment were measured and analyzed. The main research questions were:

- What colors are more visible for older adults?
- Are some colors in residential environments more attractive for older adults?
- Is gaze behavior different in older adults with normal cognition (NC) and those with low cognition (LC)?

## 2. Literature Review

### 2.1. Color and Space Perception

Color is an important factor in acquiring information because it can promote awareness and memory of the environment; however, aging vision and aging brain function can result in a tendency toward longer vision reaction time in older adults than in young adults [11,12]. For older people, chromatic pictures had an advantage over achromatic because colors are strongly felt immediately compared to form [13]; furthermore, the effect on memory lasts for a long time, so color plays a substantial role as an environmental stimulus [11].

In fact, most of the concepts of color visibility are applied to two-dimensional graphic design, including signage design in public spaces. For example, Bae et al. (2018) studied the improvement of sign color in public spaces and found that, from a distance, different color matchings have sharp and inconspicuous effects [14]. For normal people, if the background color is white, the visibility decreases in the following order: purple, blue, cyan, green, purple, red, orange, yellow green, yellow orange, and yellow. When black is the background color, the opposite is the case [15] (p.112). Usually, when matching two colors, the most prominent color has high visibility even if it is farther away [14], but the differentiation effect of space is greatly reduced when more than two colors are in the field of view [16].

Min et al. (2018) noted that, in virtual space, color can provide users with systematic and logical cognition in the architectural environment space so that forming a cognitive map of the space is easier and faster. Therefore, once the color enters the eyes, it reaches the brain to form the cognitive process of space [16], but some older people have lower cognitive abilities than younger people as a result of aging [17]. At the same time, significant differences exist in spatial cognition in older adults with cognitive impairment and those without cognitive impairment [10].

### 2.2. Physiological Characteristics of Older Adults

According to Delcampo-Carda’s literature review (2018) on spatial color for older adults, lens density increases during physiological aging. In addition, 20% of people over 75 years of age experience a change in their sense of color resulting from the yellowing of the lens [11]. Maculopathy of the eyes occurs when proteins are decomposed into amino acids as they are exposed to ultraviolet light for a long period of time, and their color changes from yellow to brown. A combination of these problems affects the absorption of short wavelengths of light, reducing the perception of short-wavelength colors such as purple, blue, and green [11,18]. Hassan et al. (2015) also discovered that in a comparative study of visual color cognition deficits in the elderly, the color seen by the elderly would be more yellowish, and there would be a certain loss of color perception [19]. The results of the comparative study show that the perception of color observed in older adults deviates from normal; for example, the blue loses its blue feel, and purple and yellow appear brown and orange, respectively [19].

In addition, older adults’ cognitive capacity decreases because of the physiological aging of the brain [20]. Cognitive capacity is also affected by external conditions, such as education level, occupation, and the social welfare system [21,22]. Cognitive problems including perception, memory and thinking disorders, chronic stress over a long period, illness, and injury can lead to serious cognitive problems. Cognitive decline due to aging is relatively weak compared with problems caused by diseases [23].

### 2.3. Eye-Tracking Technology

Eye-tracking technology measures eye movements and provides researchers with objective data on the gaze behavior of the human beings under study. It can provide information on the position, time, and order of gaze and pupil changes in the participants observed. In general, eye-tracking relies on a task assigned to a participant, but in the absence of a task, the participant naturally observes the content of the image and the surrounding spatial environment as in daily life. Areas of interest (AOI) within stimuli that “define specific regions on the page” [24] (p. 171) are usually set on the target, which is defined by a set of x-y coordinate areas divided according to the specific content or the needs of the researcher on the viewed screen; and the data in the area are obtained to grasp the observation of the participant on the area [24]. AOI can be generated “a priori or post hoc as geometric or free-form shapes around products items, or any other section of the image the researcher designers to analyze” [25] (p. 568).

Eye-tracking visualization can provide data on where and when the participants were looking. As the process of looking is automatic and natural, this type of measurement is an attractive and useful scientific tool for both qualitative and quantitative research. It allows researchers to understand participants with their non-conscious gaze processes in a harmless way [24].

Vision is a means of obtaining external information, and psychological and brain activities are reflected in the eyeballs, such as fixations (stops) and saccades (jumps or moves), so that the human visual observation situation can be effectively captured and acquired through the eye-tracker. Fixations refer to the fixed time of vision on an AOI, and saccades are movements between fixations [26]. The two main concepts addressed in this study include entry time, which is the “[a]verage duration from start of the trial to the first hit of an AOI,” and the dwell time, which is the “sum (all fixations and saccades within an AOI for all selected participants)/number of selected participants” [27] (p. 236). Dwell time can be interpreted as a measure of “cognitive processing (thought) through attention” [25] (p. 568). Data on eye movements in specific AOI allow researchers to explore human responses to stimuli. For example, using an eye-tracker, Baik et al. (2013) found that college students were more interested in high-contrast color combinations [28].

In a study by Suh and Cho (2021), visual attention in a space was related to spatial ability. Those with relatively high spatial ability paid more attention to spatial components, whereas those with relatively low spatial ability were affected by decorative visual stimuli [29]. The dwell time associated with this attention-related observational motivation was the sum of the participant’s fixations, browsing saccades, and revisits; the number of fixations was closely related to the dwell time [24,30].

The long dwell time in an AOI can indicate greater interest in the AOI [31] or the presence of information difficult to understand [32]. Entry time refers to the time when the user enters the set AOI for the first time [33]. A heat map is created to visualize the collected visual data and to gain a sense of what areas of the page attract more (or less) visual attention [24]. In the visualization the brightest areas (red) represent a greater density of fixations.

## 3. Experiment 1

Experiment 1 was conducted to identify the most visible color that would be applied to the main experiment (Experiment 2). In our prior study (Wang and Cho, 2022) we used eight color combinations that included four safety colors (red, yellow, blue, and green) to reveal that the red–black combination attracted relatively more attention from older adults than other colors, such as blue–black and yellow–white [34]. Safety colors are those associated with special meanings related to safety, that is, the standard colors used on signs in public places [35]. Only color patches were used in our earlier study; therefore, we used the standard safety colors in Experiment 1, including white–white on interior doors with nine different combinations to clarify the color scheme of the door in the space and to explore seniors’ attention to different door color schemes.

### 3.1. Research Method

#### 3.1.1. Stimuli

**Figure 1:**
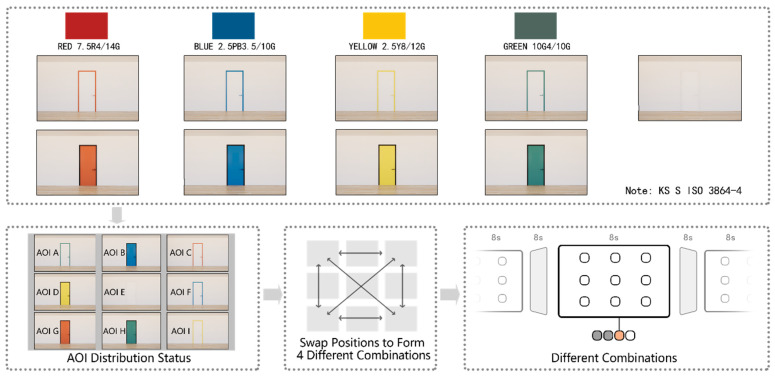
Stimuli and Procedure.

By applying four safety colors, white, and black to either the door or door frame, a total of nine different color combinations of door–frame conditions, including white–white, were generated and used as experimental stimuli (Figure 1). The experimental stimuli were generated using SketchUp and Enscape software. To reduce any influence from the position of specific-colored doors in a specific placement in the stimuli, four images with different arrangements of doors were generated and used in the experiment. To explore the attractiveness of doors of different color combinations to older adults, AOIs were set up on each door and wall. Four arrangements and nine AOIs were used at different positions as shown in Figure 1.

#### 3.1.2. Equipment and Environment

We used an eye-tracker by Senso Motoric Instruments REDn Professional set under a 24” monitor (i.e., 537.6 mm × 296.5 mm without frame). The experimental software used was SMI Experiment Center 3.5, and the experimental result analysis software SMI BeGaze 3.5. The participant’s face was 60–80 cm away from the screen based on the instructions of the manufacturer of the instrument [34]. To minimize display fluctuations in the experimental environment, display brightness was maintained at the same value throughout the experiment for each individual. The monitor and eye-tracker were connected to the same laptop, and the eye-tracking data were stored at 60 Hz [36].

**Figure 2:**
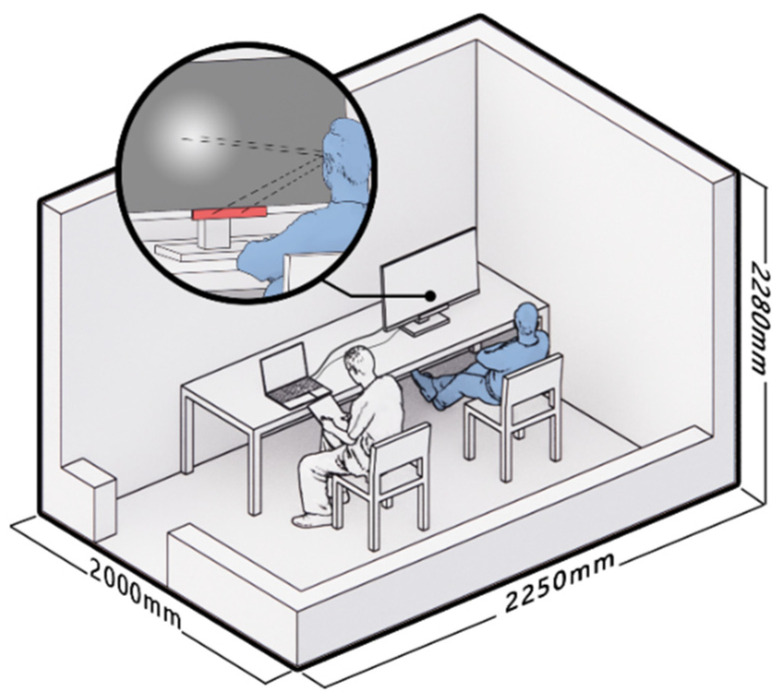
Experimental Environment.

The experiment was conducted at an adult daycare center located in Songpa-gu, Seoul, Republic of Korea. The experimental environment was a quiet room with a size of 2250 mm (width) × 2000 mm (depth) × 2280 mm (height). The room had an LED light in the ceiling with a 300 mm × 1200 mm size. The average illuminance of the room was 550 lux, and the color temperature was 4500 K. In the experimental space, a monitor with an eye-tracker and a computer were arranged; two chairs were available for the participant and the experimenter (Figure 2). Only one participant entered the room for the experiment. The experiment was carried out with a Dell 24” monitor; the screen had a matte surface, and the monitor maintained identical brightness of the screen in the experiment. The open space outside the experiment room was equipped with a laptop and two chairs, which were used by the questionnaire investigators and participants after the experiment. This study was conducted with the approval of the Institution Review Board (IRB) of the authors’ university.

#### 3.1.3. Procedure

The procedures were as follows:

1. Before the experiment, consent for participation in the experiment was obtained, written by the guardians of the older adults who participated in the experiment; the contents and procedures of the experiment were introduced.
2. A prepared numbered ticket given to each participant included participant ID and checkboxes for the eye-tracking experiment, survey, and reward. Check marks were made once they completed each of the procedures.
3. The participants entered the eye-tracking experiment room and sat in the experimental position.
4. Researchers adjusted the position of the participants and the monitor to secure the distance between the head and the monitor at 60–80 cm (65 cm is the optimal position) and to set their gaze at the center of the screen. We made sure that the participant’s chair did not have “wheels and pivots to minimize the amount of upper body movements made by the participant” [37] (p. 167). Researchers advised participants to “look at the target while keeping his/her head still as much as possible” [37] (p. 167).
5. Calibration was performed to adapt the eye-tracking software to the participant’s eye characteristics and to ensure the validity of the data collected. Calibration,“[c]hecking the accuracy and precision of the obtained eye tracking data is essential to maintain the quality of the data” [36]. The calibration result showed accuracy values that indicated deviation and eliminated unqualified data [38]. We eliminated data above 0.5 degrees based on the recommendation [38] (p. 104).
6. A training using sample stimuli was conducted so that participants could carry out the experiment with a clear understanding of the experimental procedure as well as become familiar with the setting.
7. The main experiments were conducted. The observation time for each image was 8 s; an 8-second gray blank image was added between the images to prevent any carry-over effect. Because the explanation and equipment adjustment before the start of the experiment varied depending on the condition of each participant, the average experimental time per participant was approximately 5–12 min.
8. A survey questionnaire was conducted outside the room regarding the participants’ experiences with the eye-tracking experiment. A research assistant read the questions and recorded the participants’ answers on their behalf.

#### 3.1.4. Participants

Participants were recruited from among those attending the adult daycare center regularly. The influence of coronavirus disease 2019 (COVID-19), and considering the health status of the elderly at that time, made obtaining consent from participants difficult. A total of 15 older adults, ranging in age from 68 to 89 years of age, consented to and participated in the experiment.

### 3.2. Results

#### 3.2.1. Accuracy and Precision

To ensure gaze data accuracy and precision through the calibration process, the tracking ratio of the gaze data must exceed 80%, and the deviation of the X- and Y-axes of eye-tracking should be less than 0.5 [36]. Tracking ratio means “[n]umber of non-zero gaze positions divided by sampling frequency multiplied by run duration, expressed in percent” [27,36]. Accuracy means “the difference between true and recorded gaze direction,” while precision means “how consistent calculated gaze points are, when the true gaze direction is constant” [39].

Although 15 people participated, valid data from only eight of them (four men and four women) were used to analyze the participants’ visual acuity after screening the tracking ratio and deviation data. We removed data with under 80% tracking ratio and larger than 0.5 degrees in deviation.

#### 3.2.2. Entry Time and Dwell Time

**Figure 3:**
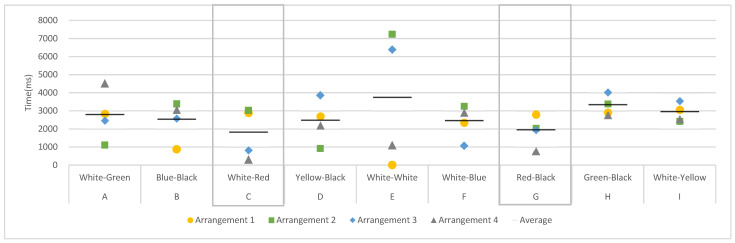
Entry Time in Different Door-Frame Combinations.

First, the entry time was analyzed by using the area of each door and wall as the AOI in four different arrangements. The entry time was the time to enter the set AOI for the first time. A smaller entry time value indicated that people viewed the AOI early and quickly. Figure 3 shows the mean value of the entry time for each AOI across the four images with different arrangements. The doors containing red (AOI-C and G) were observed faster by participants. The average entry time value of AOI-C was the smallest, followed by AOI-G. Except for AOI-E, which had the same white color as the wall and door, the average entry time was the largest in AOI-A and AOI-H, which were green-based colors.

**Figure 4:**
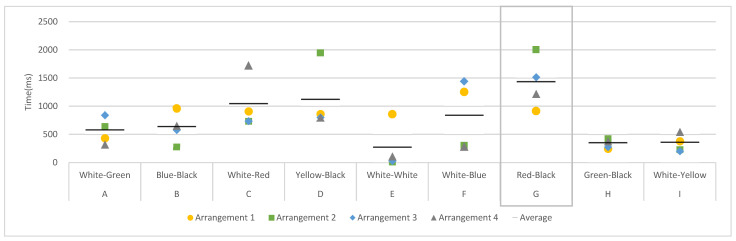
Dwell time in different door–frame combinations.

Second, to understand the participants’ attention, dwell time was analyzed. Dwell time was the value at which the gaze remains in the AOI; the larger the number, the longer the observation time. Across the four arrangements (Figure 4), dwell time in AOI-G (red-black color matching) was the largest, indicating that the participants’ observation time in AOI-G was the longest. Except for AOI-E, where white doors the same color as the wall were used, the dwell times of AOI-H (black–green) and AOI-I (white–yellow) were relatively short, which means these colors were less attractive to the participants.

**Figure 5:**
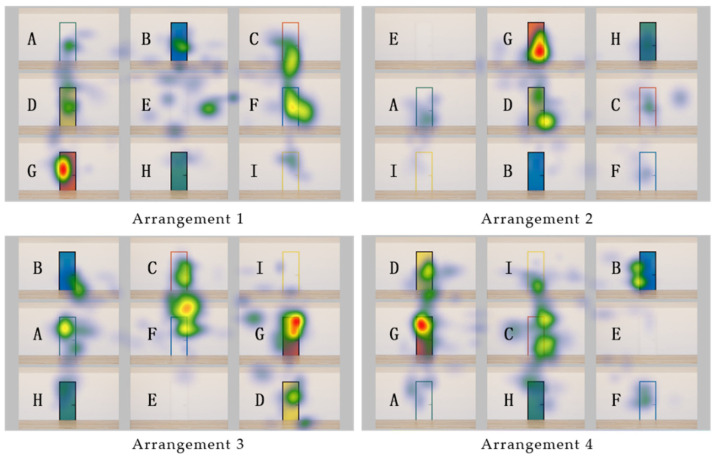
Heat map in different arrangements. Note: A ~ I: AOI names.

The image below (Figure 5) is the heat map generated from the eye-tracking data. A heat map is composed of “gaze patterns over the stimulus image visualized as a colored map” [27] (p. 104). The image shows the distribution and intensity of data occurring at the observation point. Blue indicates fewer data, and red indicates more data [40]. The heat map shows that, regardless of the arrangement of doors in each image, participants in the experiment focused more on the AOI-G (red-black) color matching than on the other doors.

The results of Experiment 1 indicate that when older adults observed doors with different color combinations, the standard safety color red was more attractive than the other safety colors, and red–black was also more eye-catching for them. Therefore, we decided to use the red–black color as a high-attention color in Experiment 2.

## 4. Experiment 2

Experiment 2 was designed to apply the observation results from Experiment 1 to a simulated living environment and examine the older adults’ gaze behavior at the door color with high visibility and with low visibility. In addition, it was intended to explore the differences in gaze behavior between older adults with normal and low cognitive states.

### 4.1. Research Method

#### 4.1.1. Stimuli and Procedure

**Figure 6:**
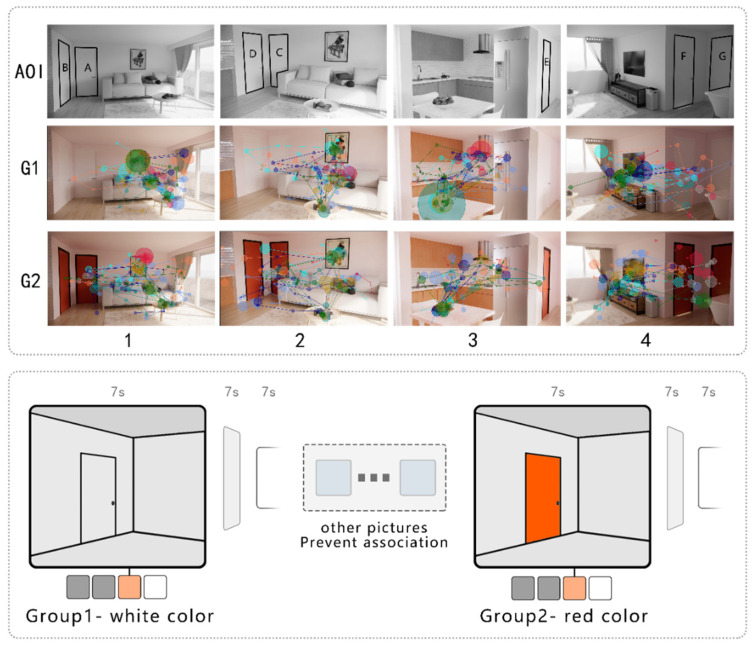
AOIs, stimuli scan paths, and procedure.

Stimuli were developed as follows:

1. A rendering of the residential indoor environment was generated with the layout of a typical apartment in South Korea using SketchUp and Enscape software. The Group1 (G1) stimuli were comprised of four images of the same layout but taken from a different camera view, where white doors were applied (Figure 6). The Group2 (G2) stimuli were comprised of the same four spatial images as Group 1, but the door color was red–black which, according to the results of Experiment 1, can maximize participants’ visual attention.
2. To ensure the science and contrast of the experiment, the two groups in this experiment had the same spatial arrangement, lighting, and camera angles, only the color of the door was different.
3. To prevent participants from contrasting the two identical sets of pictures before and after, other images were shown between G1 and G2. The observation time per photo was 7 s, and a gray image for 7 s was set between the stimuli images to prevent carry-over effects (Figure 6).

Figure 6 shows research AOIs, scan paths of collected data, and procedure.

Because this experiment was part of a study on residential space, typical home furniture and objects were used to secure realism; however, this experiment dealt mainly with the color change of the door, so the AOI was set for only the door and the door frame.

Experiment 2 was carried out with the same procedure as Experiment 1.

#### 4.1.2. Participants

The seven male and five female participants of Experiment 2 were over 65 years of age at the same daycare center as in Experiment 1. Individuals with NC and LC were recruited through the staff of the center.

After obtaining the IRB, consent from the participants and their guardians was obtained ethically.

#### 4.1.3. Equipment and Environment

The experimental environment and equipment were the same as in Experiment 1. The date of Experiment 2 was set 30 days after the completion of Experiment 1 in consultation with the daycare center.

### 4.2. Results

As in the procedure in Experiment 1, the participants needed to meet the data conditions required by the eye-tracking equipment. Various complex factors, such as short height, small eye size, loose skin around the eyes, and eye diseases, caused the exclusion of some of the older adults. To ensure the accuracy of the data, a total of nine participants’ data were analyzed. These included five women and four men, the youngest of whom was 68 years old; the oldest, 90.

The purpose of this experiment was to understand the gaze behavior of the older adults because a color variable occurred when observing the image of the simulated indoor living environment; therefore, the entry and dwell times of the AOI for the door and the door frame in each image were the main concerns in this part of the study.

#### 4.2.1. Average Entry Time and Dwell Time

**Figure 7:**
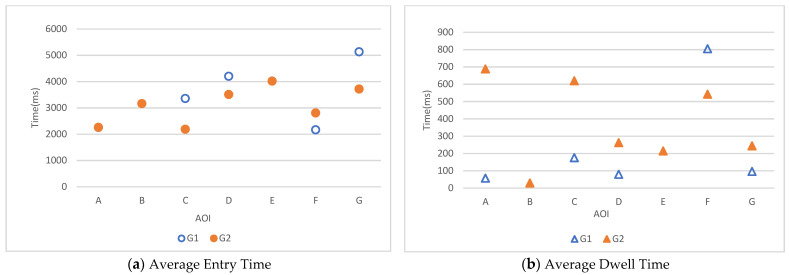
Average entry time and dwell time.

Figure 7a shows that in AOI-C, D, and G, the entry times of G2 were shorter than those in G1, indicating that, when a red door was applied to a white wall, it tended to attract a participants’ attention more quickly. In AOI-A, B, and E, only data of G2 were obtained and not G1, indicating that the white doors did not attract the participants’ attention at all.

The average score of the dwell time was analyzed and appears in Figure 7b. Results showed that in AOI-A, C, D, and G, the dwell times of G2 were longer than those for G1, indicating that when a red door was applied to a white wall, the participants tended to look at it longer. In AOI-B and E, only the data for the G2 red door were recorded.

Overall, in the stimuli using a single variable, G2 with a red door captured the participants’ attention better than G1 with a white door.

#### 4.2.2. Cognitive Ability and Eye-Tracking Data

To gain a deeper understanding of how the older adults responded to the experimental stimuli, we combined the Mini-Mental State Examination (MMSE) scores and examined the relationship between cognitive ability and gaze behavior. MMSE is a simple and effective cognitive situational assessment tool, and the evaluation ranges are as follows: a score of 24–30 means no cognitive impairment, 18–23 means mild cognitive impairment, and 0–17 means severe cognitive impairment [41]. The MMSE values of the participants are reported in Table 1. Five people had cognitive impairment, and four had normal cognitive ability. We labeled older adults with low cognition (LC) and those with normal cognition (NC), respectively.

Table 1 reports the results of each participant’s observation of the AOI. The results showed that the obtained data were quite different according to the cognitive ability of the participants. To illustrate, people belonging to NC had a better response to seeing color stimuli in G2 than LC. In the group with LC, the percentage of participants who saw any of the AOIs was 17.14% (white door) in G1 and 40% (red door) in G2, but among the group with NC, the percentages were 28.57% in G1 and 57.14% in G2. This indicated that the group with NC looked at the doors in both G1 and G2 much more than the group with LC. Participants 4 and 10 of the MMSE 12 in LC saw only two and three of the 14 doors, respectively; Participant 2, who had a high cognitive ability (MMSE of 25), saw most of the doors.

**Figure 8:**
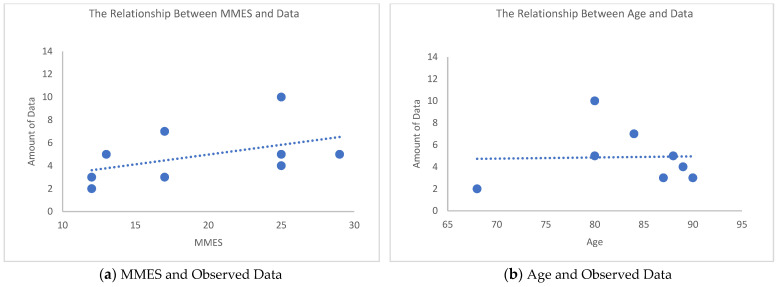
MMES, age, and the number of observed data.

Figure 8a shows the correlation between the number of times the participants saw G1 and G2 and the MMSE value. Correlation showed a proportional trend and a positive relationship between cognitive state and the number of gaze data recorded in G1 and G2 total (14 is maximum); that is, the higher the cognitive state, the more active the eye movement. Figure 8b shows the distribution of the number of gaze data recorded in G1 and G2 and the age of the participants but no relationship was apparent. Thus, we cannot conclude that the eye activity became duller with aging.

**Figure 9:**
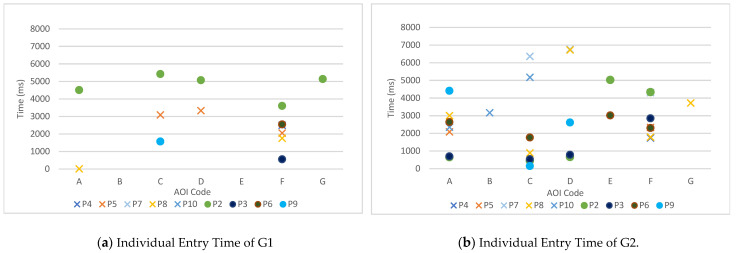
Individual entry time.

Figure 9 reports G1 and G2 entry time by each individual. O means the group with NC, and X means the group with LC.

Not all data points were obtained, so comparing the entry time within each individual for all participants is impossible; however, we can see that more data records were obtained in G2 than G1; and the data of the older adults with NC, indicated by O, tended to have a shorter entry time than those with LC, indicated by X (Figure 9a). The NC had more data than the LC; in particular, much more data accumulated for Participant 2. Among LC, Participants 7 and 10 had no data records in G1. In G2 (Figure 9b), Participant 7 saw AOI-C, D, and F, and Participant 10 saw AOI-A, B, and C. Overall, G2 data were obtained more often than G1 data.

Meanwhile, AOI-B was observed only once with the participants, which could be regarded as the least noticeable door. In the case of B and E, participants did not observe the white door (G1) at all; but when the door was red (G2), the group with NC observed AOI-E twice. Thus, the use of a more visible color seemed to play a role in high visibility.

**Figure 10:**
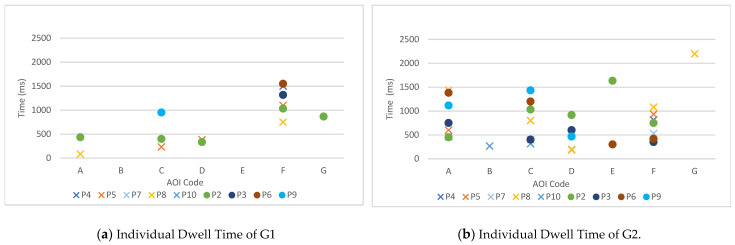
Individual dwell time.

Figure 10 reports G1 and G2 dwell time by individual.

Again, because of unbalanced data obtained from each individual, comparing the dwell time for all participants was impossible; however, we saw that the data of the older adults with NC, indicated by O, tended to have a longer dwell time than those with LC.

In the group with NC, in the data for Participant 2, AOI-A, C, D, F, and G all had data records of G1 and G2. Except for the AOI-F dwell time, the rest of the AOI data showed that G2 had a longer dwell time. Participant 3 had only G2 sight dwell time data in AOI-A, C, and D. For Participant 9, the G1 time was shorter than the G2 of AOI-C, and AOI-A and D data were only recorded in G2.

In the group with LC, Participants 7 and 10 were not recorded in G1 while Participant 7 in G2 was recorded in AOI-C, D, and F, respectively; Participant 10 in G2 was recorded in AOI-A, B, and C. Participant 8 had two data points in G1 and five in G2; and the dwell time of G2 in AOI-A and AOI-F was longer than that of G1. Participant 5 had three data points in G1 and two data points in G2. Among them, the dwell time in G2 was slightly shorter than that in G1 in the AOI-F field.

In a total of 11 cases, in one AOI both gaze data in G1 and G2 were obtained: two in AOI-A (Participants 8 and 2), two in AOI-C (Participants 2 and 9), one in AOI-D (Participant 2), and six in AOI-F (Participants 4, 5, 8, 2, 3, and 9). When one participant looked at both doors in G1 and G2, we examined their entry time and dwell time individually.

In ET, except for AOI-F, among five cases where one participant looked at both G1 and G2, in four cases (Participant 2: AOI-A, C, D; Participant 9: AOI-C) ET in G2 was shorter than in G1. This means that people tended to look at AOIs in G2 (red–black door) faster than at those in G1 (white-white door). In AOI-F, the tendency was the opposite: of six cases, two showed shorter ET in G1 (Participants 4 and 6).

In DT, except for AOI-F, among five cases where one participant looked at both G1 and G2, in all five, DT in G2 was longer than in G1. This means that people tended to look at AOIs in G2 (red-black door) longer than at those in G1 (white-white door). In AOI-F, the tendency was the opposite: out of six cases, only one showed longer DT in G1 (Participant 8).

AOI-F showing a unique tendency in gaze behavior might be the result of its size and position in the experiment image. It is the largest among all AOIs and positioned relatively in the center of the image. In addition, because of the viewpoint in the image, the door (AOI-F) is close to a TV, which has a strong contrast with the background wall. Additionally, AOI-F is located in a relatively dark position in the image, which might weaken the contrasting effect of color at G2. This indicates that when designing space with high color visibility, not only considering color itself but also size, location, and brightness are essential.

## 5. Discussion

### 5.1. Summary

The purpose of this study was to conduct two experiments to understand older adults’ gaze behavior regarding color visibility in a residential environment using eye-tracking technology. A summary of the results of Experiment 1 follows:

The average entry time of the AOI set in the red color matching (red–black, white–red) door was the smallest, indicating that red can attract more attention from the older adults who participated in the experiment.

The average dwell time of the red–black colored door was the longest, meaning that the older adults in the experiment stared longer at the red door.

A heat map is a synthesis of the acquired eye-tracking data; the older adults’ data were relatively dense and focused on red–black doors.

In Experiment 2, a red–black colored door, which was the most effective in Experiment 1, was applied to a simulated interior environment to track the gaze behavior of the older adults with the following results:

The participants had a higher interest in color optimized in G2 than in G1.

When gaze entered the AOI first, the average entry time was faster in the AOI set in G2 (red–black) than in G1 (white–white).

When both groups looked at the same AOI for an extended time, the G2 corresponding AOI was noticeably more attractive than the G1 corresponding location.

Considering the MMSE cognitive level of the experiment participants, one can say that the weaker the cognitive ability, the less active the visual observations. For the same stimulus the amount of data in the group with LC was 28.6%, and the amount of data in the group with NC was 43%.

The older the participants, the slower their eye movements. For older adults with LC, colored stimuli in the space attracted their attention.

### 5.2. Aging and Color Visibility in Older Adults

The findings from this study show that red can attract older adults’ attention compared to other colors, and red–black coloring has a better effect; this is consistent with the results of our prior color patch studies [34]. Older adults have narrowed vision and weakened discriminating ability regarding color due to the physiological aging of vision. The tendency of older adults to pay more attention to red can be explained as follows.

Effects of contrast: Participants responded faster to the red element AOI (red–black, white–red), followed by a combination of black–yellow and blue–white, characterized by a distinct contrast between the door and the wall. The vision of older adults becomes blurred during the aging process, and the contrast of objects in front of their eyes decreases as a consequence of diseases such as cataracts. Those who are visually impaired have a high interest in the stronger contrast between color, saturation, and brightness [11].

Effects of color wavelength: The problem of maculopathy in visual aging can be differentiated from the response to color in older adults and younger generations. According to Yada (2021), as one grows older, yellow pigment accumulates in the lens, turning the appearance of the object yellow, and older adults’ ability to recognize yellow decreases [3]. Hassan et al. (2015) predicted colors that would likely be perceived by people in their 70s and 80s using four color patches from the Macbeth Color Checker (a color rendition chart that has a consistent color appearance effect under various lighting conditions, especially for color checking of typical color photographic film) [19]. They compared the color results of Okajima’s method based on the Munsell color space with Tanaka’s method based on the RGB color space. The two methods produced the same results for the color perception observed by the older adults: blue loses its blue feel, and purple and yellow look brown and orange, respectively.

In addition, yellow pigment accumulation weakens the response to blue light because of denaturation. Yellow changes inside the eye make distinguishing between white and yellow difficult for older adults, and the relatively dark blue–black color also adds to the difficulty of identification. The finding that older adults were more aware of red and yellow, which are warm colors, rather than green, blue, and purple, is consistent with the results of previous studies.

Effects of the size where color was applied: As the vision narrows with aging, color discrimination on the outer periphery of the center of view is weakened. The diameter of the individual pupil becomes relatively small, reducing the flexibility of the pupil for light and shade changes. Color recognition ability decreases as in the case of older adults with blurred vision, such as myopia or presbyopia. Color information of a large area can reduce the burden of recognition; therefore increasing the area of visible color used to attract the attention of older adults is necessary [11].

### 5.3. Effect of Brightness on Color Sensation

In aging, the size of the pupil becomes relatively small, and the amount of light in the eyes decreases as in the case of the turbidity of vitreous bodies in the eyes. Consequently, in the three-dimensional color space with the color tone, saturation, and brightness, the sense of color varies as a result of a change in one condition. When light is weak, people lose color discrimination and the wavelength of the color varies depending on brightness. Davis and Garza (2002) conducted an experiment on the reading and enjoyment experience of older adults under different lighting conditions considering that aging causes pupils to shrink and less light to enter [42]. The variables in their study included illuminance, uniformity distribution, surrounding reflectance or background, and correlated color temperature. A semantic differential method was used to collect the participants’ experience and feelings. The test results indicated that the participants gave a higher evaluation on reading and enjoyment in the environment with more than 100 fc illuminance (1076.4 lux). In addition, participants evaluations were more favorable to a black background in the uniformly high illuminance condition. Davis and Garza’s study (2002) highlighted the importance of identifying appropriate brightness for task performance.

In Experiment 2, a number of similar phenomena appeared in the AOI-F regardless of whether the cognitive ability of the older adults was normal or low, and the participants’ gaze entered G1 earlier, resulting in longer attention to G1. Because AOI-F was located in a relatively dark position, the color effect at G2 might have been weakened, and when the light is insufficient, the attraction force by the color wavelength might be weakened by the effect of contrasting the dark color to the wall.

### 5.4. Cognitive Ability and Spatial Color Visibility

Cognitive ability is closely related to brain response rate, which affects the activity of the sensing organ. In Experiment 2, the amount of gaze data obtained from older adults with LC was relatively small compared with those with NC. In addition, for older adults to see elements positioned in the center of the field of view seems easier than on the edge. If too many elements are in a scene, people with LC will likely be confused and miss target elements. Considering the data of the group with LC were 17.14% in G1 and 40% in G2, whereas those of the group with NC were 28.57% in G1 and 57.14% in G2, saying that appropriate color arrangement in space can double the chance of being seen regardless of the cognition state is reasonable. Conversely, from a specific point of view, Participant 5 (MMSE13) showed interest only in G1. The symptoms and causes of cognitive impairment are diverse; thus, more concrete evidence is needed for environmental design for older adults with low cognitive ability.

## 6. Conclusions

In this study, two eye-tracking experiments were carried out on the color perception of older adults, and the safety colors that attract people’s visual attention were examined in a living environment context. In addition, their gaze behavior data were analyzed in combination with their cognitive state. The purpose of this study was to investigate the effect of color visibility in indoor environments on older adults using eye-tracking technology and to provide guidelines for interior design for older adults in the future.

The conclusions were as follows: (a) Participants paid more attention to doors with color combinations including red (red–black and white–red), and their focus remained longer on the red–black combination; (b) older adults looked at red–black doors faster than white doors in the same position; (c) the dwell time on a red–black door was longer than on that of the corresponding position of a white door; and (d) based on participants’ MMSE values, the gaze behaviors of the group with low cognition were less focused and observable than those of the group with normal cognition.

Through this study, it was possible to understand the characteristic of visual perception of older adults and to reveal the importance of the appropriate use of color in residential spaces. As a conclusion, we propose the following considerations when designing residential environments for older adults:

1. For more interest and attention, apply red–black coloration on a white background;
2. For high visibility, make the size of the target element large, and place such an element at the center of the space;
3. High contrasting colors will allow older people to obtain information faster;
4. Shade, light, and reflection should be used cautiously to avoid affecting older adults’ perception of colors;
5. Older adults with LC levels are relatively less responsive to color than older adults with NC. Designers of an environment for older adults with relatively severe cognitive capacity should, therefore, consider the use of contrasting and attention-capturing colors.

Proper use of color will strengthen older adults’ interest in safety-sensitive areas (e.g., bedrooms and toilets). The appropriate use of color and design can improve the quality of life of older adults and ease their families’ burden of caring. The results of this study are expected to be helpful in providing information on the use of colors that are easy to recognize in the design of residential environments for older adults.

The limitations of this study are as follows:

First, this study was conducted based on a simulated residential space to create a relatively realistic spatial atmosphere; therefore, objects other than color variables were included in the stimulus and these could have affected the participants’ observation.

Second, we used simulated images as stimuli, which may have caused a less immersive experience for participants. Originally, we planned to use simulated reality for a more immersive experience, but with the inconvenience and uncomfortableness of installing VR devices on heads and the risk of possible injury as indicated by a pilot test, we decided to use still images.

The third limitation involves a small amount of analyzed data. The COVID-19 pandemic situation and concerns about physical contact and cognitive status interfered with the recruitment of older adults as participants compared to those belonging to other age groups. In addition, the vision conditions of some participants caused a failure in data collection because the collected data did not meet the validity criteria of the tracking ratio and deviation level. However, considering the lack of eye-tracking research on older adults and the need to and importance of studying this age group in this aging society, we decided to collect as much data as possible on older adults while also considering the ethics in research. Studies on eye-tracking of older adults are sparse [43]; thus, while the experimental participants in this study were few, we believe their participation could enrich the research field and provide data for subsequent research.

In addition, it was challenging to replicate a laboratory environment such as by controlling wall reflections and lighting conditions because the research was conducted in a daycare center. According to Royer et al. (2021), the study made a detailed workflow for exploring the experimental environment and experimental process of color, which also provides a reference for our future research. Future research will address these issues [44]. 

## Figures and Tables

**Table 1:** Individual sight distribution.

MMSE		Participant ID	Age (2021)	Sex	A		B		C		D		E		F		G		Total
					G1	G2	G1	G2	G1	G2	G1	G2	G1	G2	G1	G2	G1	G2	
LC	12	4	68	M											◯	◯			2
	12	10	90	F		◯		◯		◯									3
	13	5	80	M		◯			◯		◯				◯	◯			5
	17	7	87	F						◯		◯				◯			3
	17	8	84	F	◯	◯				◯		◯			◯	◯		◯	7
NC	25	2	80	F	◯	◯			◯	◯	◯	◯		◯	◯	◯	◯		10
	25	3	88	M		◯				◯		◯			◯	◯			5
	25	9	89	F		◯			◯	◯		◯							4
	29	6	80	M		◯				◯				◯	◯	◯			5
Total					2	7	0	1	3	7	2	5	0	2	6	7	1	1	

Note: G1: Group1; G2: Group 2. LC: Low Cognition; NC: Normal Cognition. ○:data is recorded.

## Data Availability

Not applicable.
